# Barriers and opportunities to using health information in policy implementation: The case of adolescent and youth friendly health services in the Western Cape

**DOI:** 10.4102/phcfm.v13i1.2654

**Published:** 2021-02-25

**Authors:** Myrna van Pinxteren, Sara Cooper, Christopher J. Colvin

**Affiliations:** 1 Division of Social and Behavioural Sciences, School of Public Health and Family Medicine, University of Cape Town, Cape Town, South Africa; 2 Cochrane South Africa, South African Medical Research Council, Cape Town, South Africa

**Keywords:** policy implementation, health information, youth health services, health systems, sexual reproductive health, health service strengthening

## Abstract

**Background:**

The production, use and exchange of health information is an essential part of the health services, as it is used to inform daily decision-making and to develop new policies, guidelines and programmes. However, there is little insight into how health care workers (HCWs) get access to and use health information when implementing new health programmes.

**Aim:**

This study explored the multifaceted role of health information within policy implementation processes and aimed to understand the complexities experienced by HCWs who need to develop adolescent health profiles (AHPs), a criterion of implementing a larger Adolescent and Youth Friendly Services Programme (AYFSP).

**Setting:**

This case study was undertaken in Gugulethu, a peri-urban, low-income neighbourhood in Cape Town, South Africa.

**Methods:**

Data were collected through ethnographic qualitative methods, including participant observation, interviews and workshops, and 15 participants were enrolled for this purpose.

**Results:**

Findings showed that HCWs experienced different barriers when accessing information to develop the AHPs, including a lack of access to databases, a lack of support and inadequate guidelines. Nevertheless, HCWs were resourceful in using informal information and building strategic relationships to navigate and gain access to the necessary data to develop AHPs.

**Conclusion:**

This case study provided insights into the practical difficulties and innovative strategies which arise when HCWs attempt to access and use health information within a real-life health programme. Findings highlighted the need for more training, support and guidance for HCWs to improve the meaningful use of health information during policy implementation processes and to strengthen health services in South African primary care clinics.

## Introduction

### Background

The production, use and exchange of health information is an essential part of the health services and is one of the six World Health Organization (WHO) health system building blocks.^1^ Health information is used to inform daily decision-making in the health services, but is also used to develop new health policies and guidelines to improve the quality of care and access to these services.^2^ Whilst the role of health information in the development of policies and guidelines is recognised, including in South Africa, the need for, access to and use of health information during the implementation process of new health programmes and policies is less appreciated and understood, particularly in low-and-middle-income countries (LMICs).

The few studies that have been conducted on this topic revealed that there is a need for and interest amongst health care workers (HCWs) to use health information during policy implementation processes. However, these studies did not report if and how health workers were able to access this information in practice.^3,4,5^ This is a pertinent gap in research, as health information is important in daily decision-making in clinics as well as plays a role in operationalising health programmes to strengthen health services.

At the same time, in LMICs including South Africa, most research conducted about health information focuses on how routine information is collected, stored and communicated through dedicated health information systems, including patient records, facility surveys, routine management reports (RMRs) and disease surveillance reports.^2,6,7^ Although understanding the collection and use of routine health information is important, there is an array of ‘informal’ or ‘soft information’ that is often not recognised as being important in the health system.^8,9^ This is a shortcoming, as health actors including managers and HCWs use different forms of information for decision-making, both within daily practice and when implementing new programmes and guidelines. Indeed, policymakers, both local and national, use a combination of formal and informal evidence when developing new policies or programmes.^2,6,7,10^ Sources of information include international guidelines and academic research as well as relevant local health services data.

This research article, which is based on a qualitative case study, explores how a group of HCWs in Cape Town, Western Cape, negotiate access to different types of health information and use and exchange this information during the process of developing an adolescent health profile (AHP), one of the requirements of the larger Adolescent and Youth Friendly Services Programme (AYFSP) that seeks to strengthen the health services for young people in South Africa.

This article provides insights into the practical difficulties, as well as innovative strategies, which arise when HCWs are tasked with working with health information within a real-life policy implementation process. We also show the multifaceted and complex role of health information within policy implementation processes and highlight the importance of accessing both routine and informal forms of health information.^11,12^ We furthermore emphasise that health information is often seen as a ‘black box’, whereby the importance of evidence is recognised, but we fail to understand the day-to-day access to and use of information. Based on the findings from the study, we also provide recommendations for operationalising new health programmes going forward and how health information might be more effectively used in this process.

### Adolescent and Youth Friendly Services Programme and adolescent health profiles in South Africa

The AYFSP is a dedicated programme that aims to strengthen the quality of, and access to, public health services for young people in South Africa, which was developed in 2017. The programme is linked to other initiatives to improve and prepare primary health facilities for the planned National Health Insurance (NHI) and the larger Ideal Clinic Realisation and Maintenance (ICRM) programme that is currently being rolled out across South Africa. The programme has a particular focus on sexual and reproductive healthcare and is embedded in the National Adolescent and Youth Health Policy.^13,14,15^ Through the implementation of the AYFSP in clinics, young people should receive better care as a result of shorter waiting times in clinics, flexible opening times of clinics and receiving health education and health information through mobile apps.^16^

There are several criteria that need to be fulfilled when implementing the new AYFSP and becoming a ‘youth friendly’ clinic. One criterion is the production of an AHP, a document which is kept in the clinic and contains information about the lives and challenges of young people in the catchment area of the clinic.^14^ In South Africa, very little is known about the requirements for, and effectiveness of, AHPs. In other countries, including the United States and the United Kingdom, AHPs or community health profiles (CHPs) are developed to underline and explore how social determinants of health impact well-being and health-seeking behaviour in communities. These social determinants of health include racial segregation, poverty, poor access to health services and high rates of violence – which differ between countries, but also affect individual neighbourhoods.^17,18^ Although the information needs to be accurate, complete and up-to-date, CHPs are often seen as ‘conversation starters, highlighting issues that can affect health in each locality’.^19^

To develop an AHP as required for the AYFSP, clinic staff are encouraged to use different sources and databases to collect demographic information about the youth in their area and to map the availability of, and need for, sexual and reproductive health services in both the clinic and the community.^14,15^ By doing so, HCWs learn more about the issues that young people face that impact their health decision-making. Ideally, this leads to health workers anticipating the needs of young patients when they come to the clinic, potentially resulting in better provision of health services and improved health outcomes for this vulnerable group of people.^14^ Once all the information is collected into a comprehensive profile, this document is kept in the facility for internal use and updated every 2 years with new health information. Setting up the AHPs and the implementation of the rest of the AYFSP is often the responsibility of clinic managers, who directly report to their sub-district and district managers, or ‘Champions’ – appointed HCWs who have a passion for or expertise in specialised health services, such as youth healthcare programmes.^20^ During this process, they are assisted by implementation partners appointed by the Western Cape Department of Health (WCDoH).

Creating an AHP is a valuable initiative to better understand the social realities, lives and health challenges of youth, which can potentially inform the improvement of health services for young people. However, within the AYFSP, specific guidance on how the AHP should be developed and what information would be needed to populate this document was not provided, which caused confusion amongst HCWs and implementation partners.

This case study aims to unpack some of the issues that HCWs experienced when trying to develop these profiles as a way to better understand how health information is accessed and managed in the context of implementing a youth health programme. Through the use of ethnographic methods, we identified a range of barriers that hindered HCWs to successfully create AHPs. Specifically, there is a persistent misperception around the access to and use of health information within clinic settings, as there is often too much emphasis on the production of health information for reporting and management purposes, but very little recognition of the practical difficulties of accessing and using information for daily decision-making in facilities. By following HCWs on their journey to gather the necessary information to develop their AHP, we are able to surface some complexities about health information use and exchange, show the resilience and creativity of health workers when negotiating access to information and unpack the role that informal information plays within daily decision-making and when operationalising new health programmes.

## Methods

### Study design

This article is part of a larger qualitative study which focused on the complexity of health information use and exchange amongst health systems stakeholders and community actors in Cape Town, South Africa.^21^ The article is based on one of three case studies, which is defined by Robert Yin as ‘an empirical inquiry that investigates a contemporary phenomenon (the “case”) in depth and within its real-world context’.^21^ The broad aim of the case studies was to understand how research, community activists and health workers accessed and used health information to improve health services in Gugulethu. For this article, we followed a group of 15 HCWs on their quest to implement the AYFSP and used qualitative methods to understand how they navigated the process of accessing and using health information to develop the AHPs. The HCWs were recruited through relationships with key informants and convenience sampling during a workshop. This ethnographic study was in turn nested within the iALARM project (Using Information to Align Services and Link and Retain Men in the human immunodeficiency virus [HIV] Cascade), a 5-year research project that aims to synthesise, collect and distribute health information to health system and community actors as a health system strengthening initiative.

### Study population and sampling strategy

For this artilce, the first author (MvP) followed a group of 15 HCWs on their quest to collect the necessary information to develop AHPs as part of the AYFSP implementation process. All participants were working in the Klipfontein/Mitchell’s Plain health district, which is comprised of several low-income neighbourhoods (townships). Participants were recruited through the snowball-sampling recruitment and strategy whereby the researcher (MvP) established a close relationship with one HCW, Sister Maria,^1^ who introduced her to other HCWs who were collecting data for the AHPs. At the time of data collection, Sr Maria worked as a facility manager in a satellite clinic in Gugulethu. During an informal interview, Sr Maria spoke to us about her challenges with collecting the necessary health information for the AHP. Other participants were recruited during a workshop which was organised by the iALARM team to assist HCWs with the development of AHPs as part of AYFSP implementation process. Participants for this study were all employed within the public healthcare system and worked in community health clinics (CHCs), satellite clinics or day hospitals (clinics) and are directly or indirectly linked to the iALARM project. The detailed encounters with HCWs are described in the ‘Findings’ section.

### Data collection

Data were collected in 2017 and 2018 and comprised several different qualitative tools, including formal and semi-formal interviews, field notes written by MvP and a reflection of the AHP skills-building workshop which was organised in February 2018. The ongoing relationship between MvP and HCWs that she established during her 18-month period of intensive fieldwork for her PhD provided an opportunity for frequent conversations, reflections and discussions which were iterative in nature. Additional field notes and reflections on the implementation process of the AYFSP were collected by MvP, as she attended several meetings from both the WCDoH and the National Department of Health (NDoH) and spoke to several non-governmental organisations (NGOs) who are assisting HCWs with the implementation of the AYFSP.

### Data analysis

All interviews with individual HCWs were conducted in English and transcribed verbatim. Added to the interviews were field notes written by MvP about her experience working with HCWs, attending the meetings at the WCDoH and the NDoH and organising the workshop. Together, the notes and interviews were analysed through thematic analysis, an iterative process which was conducted in Nvivo11. After similar concepts were explored, several themes emerged which were divided in different barriers and opportunities. To ensure reliability of the collected findings, we used triangulation, collecting data from different sources, including interview transcripts, field notes, meeting minutes and minutes taken at the workshop. We also engaged in ongoing conversations with key informants who provided feedback and interpretation on the data collected to mitigate biased views from the research team.

## Ethical considerations

All participants for this study participated on a voluntary basis and gave either written consent or verbal consent where written consent was not possible or appropriate. All interview transcripts, recordings and field notes were stored in a password protected computer with facial recognition. Ethical approval for this research was obtained from the Human Research Ethics Committee (HREC) at the University of Cape Town (380/2017), as well as from the WCDoH and the City of Cape Town (CoCT) Department of Health under the larger iALARM project (802/2014).

## Findings

### Development of adolescent health profiles in Gugulethu

The iALARM team first heard about AHPs when a clinic manager, Sr Maria, approached MvP for assistance during an interview in early 2017. Sr Maria was tasked with operationalising the AYFSP in her clinic and had already successfully implemented several components of the intervention in the facility, but struggled with criterion 1.1, that is, ‘develop an AHP, which needs to be updated every 2 years’. This AHP should map the youth’s sexual and reproductive health but also include the current socioeconomic challenges and opportunities that impact the health of young people who come to the clinic.^21^ Sr Maria posed this particular request at the Task Team meetings which were organised by the iALARM team. At these meetings, a diverse group of health and community stakeholders would come together to discuss ways to better link men to HIV care as well as to promote HIV information sharing within Gugulethu. During these meetings, the iALARM team would provide HIV reports and conduct discussions about the health information needs in the community.^21,22^ Sr Maria used this Task Team meeting to plead to fellow health workers to assist with the process of setting up an AHP for her facility. Her request led to an interactive brainstorm amongst participants who were willing to contribute to the AHP. However, there was a lot of uncertainty on what exact information was needed to develop this profile that aimed to map out the health needs of young people in the community.

In the weeks following the Task Team meeting, Sr Maria and MvP worked intensively together to map out what kind of data were required for the profile, where these data could be found and who needed to be approached to get access to the various sources. Even though this sounded like a straightforward request, the reality proved to be more complicated, as the iALARM team and Sr Maria experienced many hurdles along the way. In initial conversations to map out the specifics required for the AHP, Sr Maria spoke extensively about the lack of computers in her clinic, showed hand-written patient files and shared documents with MvP that could be used for AHP. These sources were useful, but not complete enough to compile a complete profile. For the first drafts of the profiles, we relied on RMRs provided by Sr Maria, combined with general information about services, schools and NGOs in Gugulethu, Nyanga and Phillipi. The statistical data were collected through www.youthexplorer.org.za., a web-based application created by University of Cape Town’s (UCT) Poverty and Inequality Initiative and OpenUP, an organisation that provides free government statistics to communities and provides data on young peoples’ health status, schooling and living conditions.^23^ We also used data from the South Africa Police Services (SAPS) to outline young people’s experiences of crime and violence in their communities.^24^

The first draft of Sr Maria’s AHP was presented in the following iALARM Task Team meeting and although not being complete yet, received a lot of praise. After the meetings, staff members from various clinics put in requests to the iALARM team for assistance setting up their profiles, as they also received little guidance from their line managers when attempting to create their AHPs. We honoured these requests by organising focus groups with HCWs and by setting up an interactive workshop which allowed us to provide hands-on support for 20 HCWs who had the responsibility of developing the AHPs in their own facility. The iALARM team also connected HCWs with the various NGOs and implementation partners to facilitate ongoing support structures. Through these numerous interactions with HCWs, we learned a series of lessons about the complex role of health information in the context of implementing the AYFSP, the barriers faced by HCWs and how they were able to innovatively use formal and informal health data to populate the profiles. A number of insights were gained from the implementation process of the AHP.

### Lack of knowledge of and access to routine health information to create adolescent health profiles

Health care workers reported being frustrated by having little access to sources where the required health information could be found. Many did not interact regularly with databases in their clinics, as this was not part of their daily work. Therefore, they had no idea where even to begin gathering the necessary data for the profile and were not provided protected time away from their daily responsibilities to work on this requirement. Furthermore, most of the needed information was not stored in facility databases, but had to be obtained from various sources:

‘I need to make a whole community profile, can you imagine, it is something like eight or nine pages with things I need to fill in. When do I even have the time to do that, to collect all the information? I need to go into the community to get the information that I need and that takes time. I could really use assistance from someone and I know it needs to be done, otherwise I cannot officially ask for funding for the clinic.’ (HCW 1, satellite clinic, female)

Another HCW asked the following:

‘Morning, Happy New Year. is it possible Myrna that you can send us the community profile, as ours just disappeared. I don’t know how to make one, please can you come and sit with management to explain how it works.’ (HCW 2, CHC, female)

The message, which was communicated via SMS to MvP, showed that often health workers had no idea where to even begin gathering the information for the AHP. This was interesting, as the iALARM team does not specialise in either youth health services or health profiles, but MvP was nevertheless considered to be an expert in this field.

Additionally, some clinics in the area were not connected to central databases and used handwritten folders to capture patient information. This information was compiled into an RMR and sent to the sub-district manager on a monthly basis:

‘All patients have handwritten folders which are captured at reception. That is how we do our data capturing. This clinic has computers, but we are not connected to the central data system from the WCDoH, as we are officially a satellite facility.’ (HCW 1, satellite clinic, female)

### Lack of support

Apart from not being able to access databases to attain the required health data for the AHP, participants also expressed that there was little interest from line managers to support the development of the AHP:

‘So, they [*the WCDoH*] want me to create this profile, but I have no idea where to get the statistics from. They are not willing to give me anything, and the stats that I have from my own clinic are not complete enough. The complication is that my clients come from all over Cape Town and even further than that. They have different backgrounds and languages, which makes things very complicated. So, I would like to get stats from the whole of Klipfontein and Manenberg. I went to Stats SA, but they could not help me either.’ (HCW 3, satellite clinic, female)

Another health worker shared the same concerns about the lack of support from line managers and her senior colleagues in clinic:

‘We are on the same boat as other nurses. We also need to make this AHP. We have the assistance of a peer navigator who is closely connected to the youth that comes to the clinic, but we are still training him and would love to have some assistance.’ (HCW 4, clinic, female)

The AYFSP is being operationalised by the WCDoH, who appointed several implementation partners, including government organisations such as LoveLife that employed ‘Groundbreakers’ in clinics to assist with youth health service provision and provided training for the HCWs as well as local NGOs specialising in youth programmes.

Although often with the best intentions, these NGOs were not always knowledgeable about the different aspects in the process and often lacked the expertise to work with health information:

‘I have not worked a lot on the AHP yet, as we are still trying to sensitise the clinics around it. Clinic staff are supposed to create the AHP by themselves, but they do not have the time. They feel that it is a lot of work for them. And they still need to do their clinical duties. So, they end up looking for people who do it for them, because they cannot get the accurate information. Because one of the clinics I worked with they had old information. Not current data we want in the AHP. This needs a lot of research.’ (Implementation support 1, NGO, female)

A representative from another NGO also commented:

‘We tried to speak to the sub-structure if they could help us, and they do not have it, and they threw the ball back to us. And I even tried to ask people in my own organisation, but even they did not give me an overview of the HIV data in the area. If we can get help on it, we are on a highway to accreditation.’ (Implementation support 2, NGO, female)

These examples show a lack of support for health workers in their efforts to create an AHP. Findings also reveal that even amongst implementation partners, there was not enough expertise and knowledge about this particular requirement of the AYFSP. Findings highlight that HCWs understood the information to be important in the accreditation process, but did not consider where this information might be, who might have access to it, what support would be required and how much time the development of the AHP would take.

### Inadequate and ambiguous guidelines

Another theme that derived from the findings was the lack of guidelines and templates available to assist health workers with the development of their profiles. The absence of these guidelines led to ambiguity around what type of information or level of detail was mandatory for the AYFSP certification:

‘If there was a template, everyone would be able to do this. That is where the problem lies. I have been saying this for a while and have been to municipal offices where they have some demographic information for the Gugulethu and Nyanga area, but that is not enough for the health profile.’ (Implementing partner 2, NGO, female)

For the iALARM team, it took several months and multiple appointments with NGOs and health managers to figure out that the NDoH had developed a guideline, which was not widely or evenly distributed amongst implementers. This highlights that the implementation process was not streamlined and reconfirms the assumption that health information is important, but the use of health information in real-life situations is poorly understood. The guideline stipulated that the AHP should include data on adolescent sexual and reproductive health, support services for your people in and around the health facility and list the most pressing challenges that youth faces in the community. Although the guidelines gave some direction to health workers, there was still a lack of clarity on the length and level of detail that was required, even within the NDoH:

‘There are no examples, as all the clinics will interpret the AHP in their own way. We tick off the AHP when we are in the clinic for accreditation, and then the AHP stays in the clinic. I think it is good what you do to share some knowledge and provide support for individual clinics to work together, but we do not have any examples that we can help you with.’ (Implementation partner 3, NDoH, female)

Although we can only speculate why there were no examples available, the participant explained that the information in the AHP should summarise the health and health challenges of young people within the catchment area of the clinic, which varies per facility and is largely context-specific.

For many participants, the lack of a template and clear guidelines appeared to be the biggest hurdle to overcome when attempting to develop an AHP, as they did not have a supporting documentation that could guide them through the process of accumulating the right data for the profile. More broadly, this observation reflects the absence of a critical analysis of the practicalities when setting up AHPs as part of the AYFSP, as well as a general lack of coordination and communication between the NDoH, local government authorities, implementation partners and individual HCWs and ‘Champions’ during the implementation process.

In summary, these findings reflect that although there was a buy-in from HCWs to develop AHPs as part of their AYFSP requirements, this process was complex, messy and less straightforward than anticipated, mainly because of the poor access to data and lack of training provided. Additionally, the HCWs’ experiences reflect a poor operationalisation of policy ideas and point to a fragmentation of health information use and exchange which is partly caused by data being stored in different databases, which are difficult to access.

Despite the strains and barriers that HCWs faced, they proved to be resilient, resourceful and creative in navigating these challenges when trying to create AHPs as part of the AYFSP. This resulted in HCWs creating strategic relationships with other health stakeholders and partners outside of the health system as well as the active use of informal data to populate the profiles.

### Strategic relationships

The lack of data and access to the right databases to develop the necessary AHP provided an opportunity for health workers to create new relationships and strengthen existing relationships with colleagues, patients and community members. For example, the researchers from the iALARM project received numerous requests to assist with or compile (parts of) the AHP for individual clinics. Although the researchers were unable to respond to all these requests, they provided support by organising interactive brainstorm sessions and workshops.

During an interview, one HCW shared that her clinic did not have space to create a separate waiting room for the young people, so she would sit with them under a tree in the yard of the clinic and have discussions about their health challenges, experiences in the clinic and the improvements that they would like to be implemented to make the clinic more youth-friendly. Over time, she created a relationship with these young people and would even send them out into the community to collect contact details of schools, NGOs and youth programmes. All this information proved to be valuable source for her clinic’s AHP, as well as the implementation of other parts of the AYFSP.

Other participants spoke about their relationships with data capturers or clinic managers to get easier access to information or databases. Sometimes, health workers would try to keep the relationships exclusive, afraid to lose the privileges to work together with managers in the sub-district or academic partners. One health worker who received support from the iALARM researchers, commented:

‘We need to make some improvements together and only if mine is approved, I will give your phone numbers to others and you can assist other clinics. But they [*the nurses*] are dying to meet you. But please promise me that you help my clinic first, as we really need all the help we can get.’ (HCW 1, satellite clinic, female)

Many of the participants acknowledged that collaborating with colleagues, managers and the wider community was a way to gather information which might otherwise have not been available:

‘We need to collaborate, work together, and share our experience in workshops like these ones.’ (HCW 6, CHC, female)

By actively engaging with other stakeholders inside and on the periphery of the health system, HCWs negotiated access to health information from sources which would otherwise not be available to them.

### Informal and non-routine information

Besides creating new relationships inside and outside the health system, health workers also used different forms of informal information to populate their AHP, especially when routine information was not available. This informal information, or ‘soft’ information, includes non-routinely collected data which often sits in conversations, is based on personal experience and relies on HCWs’ knowledge of health services and the health system as a whole.^25,26^

Sometimes, participants would use conversations with young patients and community members as sources of information for the AHP. This information was drawn from discussions about preferred contraceptive use or how to negotiate safe sexual practices with intimate partners. Health promoters, who work both in clinics and reach out to community members, also proved to be invaluable stakeholders in the AHPs’ development process, as they were able to share information about schools in the neighbourhood, would speak to school nurses on a regular basis and interacted often with young people outside the clinic setting. Sr Maria and others relied heavily on the expertise and knowledge shared by health promoters and other colleagues, especially when gathering information about health challenges experienced by young people.

Other types of informal information were referrals to other clinics for procedures such as voluntary medical male circumcision (VMMC) or termination of pregnancy (TOP). These referrals were logged by HCWs to track young patients, but not always captured in existing clinic databases. Nevertheless, these data were mandatory for the profile, even though participants initially did not consider this information to be routine. This type of information also included stock lists of condoms and other contraceptives, which became sources of data that were used to populate the AHP.

One of the participants mentioned that she started a WhatsApp group with young female patients in the clinic. She would use the WhatsApp group to remind the girls about their appointments and tell them when to pick up their contraceptives, but also to converse with them about familial challenges, give advice about love and relationships, and listen to the concerns her young patients were sharing. For the AHP, she summarised the experiences of the young people, in order to provide an overview of the most pertinent challenges for adolescents in the catchment area of the clinic.

The brainstorming sessions during the workshop for HCWs organised by the iALARM research team (RT) proved to be a great resource of informal information for the development of the AHPs. During the interactive workshop, participants spoke about how to extract data from RMRs, were taught how to access and use the YouthExplorer platform but also offered a slot to discuss the consequences of social and economic challenges on the lives and opportunities of young people in Cape Town. Topics discussed in this slot were gangsterism, substance abuse, domestic violence, poverty, crime, HIV, sexually transmitted infections (STIs) and unwanted pregnancy – issues that young people in the Klipfontein sub-district face on a regular basis. After the workshop, the iALARM team shared a summary of the discussion with participants which could be adapted to the clinic context and included in the AHP. During the workshop, HCWs revealed that it is easy to be desensitised to these issues when hearing the same stories in the clinic every day. Naming, unpacking and discussing the impact of these challenges during the workshop proved to be useful sources of non-routine information that could be added to the AHPs.

Although not considered as routine health information, these findings show how informal or non-routinely collected information became an important source of data for HCWs when populating the AHP, as this information would fill in the gaps where routine information was not available. Sharing stories and creating open dialogues and discussions amongst participants proved particularly useful during this process, as this provided an opportunity for relationship building, both amongst participants and between HCWs, managers and implementation partners.

## Discussion

This study highlights several practical difficulties, as well as innovative strategies, which arise when HCWs are required by their line managers to work with health information within a real-life policy implementation, which is operationalised through a top-down process. Experienced barriers amongst participants were the lack of access to databases and other relevant sources of data, limited guidance from managers and implementation partners and the absence of clear guidelines and templates. Besides the experienced barriers such as the access to information, the case study also shows that HCWs are creative and resilient when trying to collect the necessary information for the AHP. Hereby, they strategically use relationships with colleagues, managers or academic partners, but also collected different forms of non-routine data to fulfil the proposed requirements.

The findings from this study also reveal insights about the multifaceted and complex role of health information within policy implementation processes. It highlighted how policymakers and implementers know very little about the day-to-day use of health information and have limited knowledge on how, when and under which circumstances HCWs are accessing and using health information. Furthermore, it emerged that there is a simplistic understanding by both policymakers and health managers on what health information actually is, where this information can be found, who is able to use it and how it is used within the intervention space. That is, health information was treated somewhat like a ‘black box’, whereby the importance of the evidence is recognised, but there is a failure to understand the day-to-day access to and use of this information. Moreover, whilst the AHP was identified as a requirement of the AYFSP, no explanation was provided about the origin of these profiles, how they were used in other contexts and why it is important to create AHP to improve youth health services, which led to confusion amongst participants.

What emerged in this study was how health workers are using both routine and informal forms of health information to develop the AHP as part of the AYFSP and how participants used this information effectively to fulfil the requirements of the programme to improve health services for young people. Although informal data is often underacknowledged, as it is harder to measure and transfer, it does inform daily practice and decision-making in health services.^8,26,27^ Results from this study therefore feed into a larger debate about the role of information in policy implementation processes, but also highlights that we know very little about how HCWs interact and engage with information, as they are often left out of data management conversations which take place elsewhere in the health system.^25^

This study also speaks to the messy and complex process of policy implementation more generally. Successful policy implementation frequently depends on the buy-in of those who are putting the policy into practice, as suggested by Erica Orange, Beland and Ridde who argue that implementers are less likely to dedicate time to implement new programmes in their already busy schedules if they are not actively involved in the development of the policy. Health workers and champions may have different ideas on what to include in the policy and what is feasible within their facility, but they are rarely consulted in the writing of the policy.^28^ This is especially true for vertical health programmes, which are often informed by international guidelines which are adapted for a local setting, without always taking into account local realities.^29^ Additionally, when the implementation process is poorly organised and the interest of health workers is not taken into account, it further hinders the successful implementation of new guidelines or programmes.^28,30,31,32^

In this case study, the access to health information was underestimated by policymakers, and the different types of information required were poorly understood. Health profiles can be very useful to predict the health needs of specific populations within communities, but also require a huge amount of information organised systematically for it to have meaning for HCWs.^19,33^ Therefore, to be successful, the necessary resources and infrastructure need to be made available and the information should be translated into an accessible format. Furthermore, trying to standardise the requirements of CHPs or AHPs is impossible, as indicators and available data vary drastically across contexts. The lack of guidelines and available infrastructure significantly hindered HCWs’ ability to effectively create an AHP, as most of the participants had no idea about where to start the process. Although this is understandable, this also led to frustration, especially amongst some facility managers who translated barriers experienced by HCWs into the unwillingness to develop necessary profiles.

The unclear guidelines and lack of an available template point to a disconnect between the design of health programmes and how they are operationalised in real-life settings. Additionally, this disconnect emphasises the complex role of health information that is simultaneously under- and over-presented. On the one hand, policymakers predict that the use of health information, in this case in the form of an AHP, will solve part of the multilayered puzzle by informing health actors about young people’s issues and the type of care they need. On the other hand, the lack of details in the guidelines also reveals that there is little recognition of how complex the access to and use of health information can be.

Although this case study gives an in-depth understanding on how health information is accessed and used by HCWs to develop AHPs as part of the AYFSP in the Western Cape, some limitations were observed in the process of data collection, as our participants were all in different stages of developing the AHP and did not have equal access to resources, which caused confusion during the interviews. Although we did participant observation at the workshop, we didn’t have a chance to conduct in-depth or longitudinal interviews with all participants.

## Recommendations

Although this case study shows only a snippet of the implementation process of the youth health programme in clinics in South Africa, some recommendations can be made to assist policymakers, implementation partners and HCWs to optimise the operationalisation of new health programmes going forward and how health information might be more effectively used in this process.

Recommendations include the need to provide clear guidelines and usable templates that can be used when collecting the needed health information and to develop strategies to support HCWs during every step of the implementation process. These strategies should include training and discussion sessions about new ways of accessing, coordinating and using both routine and informal health information. Furthermore, dedicated time should be provided to health workers to search for, assess and synthesise health information under the guidance of professionals who are able to clarify when and under which circumstances evidence can be activated to assist decision-making processes. Another strategy which can be considered to streamline the process of policy implementation is to actively engage frontline HCWs during the policy development process. Health care workers can give input on what information is crucial to improve services within their clinics and can tell policymakers if they have access to this information and what data are missing. Health care workers can also assist with adapting generalised data – such as a population or census data – to their local context, as they are experts in their own clinics and know their patients and health challenges. Insights from this case study may help to inform new ways of thinking about the multifaceted role that both routine and informal health information play in health programme implementation in South Africa and how different forms of health information can be used meaningfully to strengthen service delivery and the health system at large.

## Conclusion

As South Africa is currently moving towards a more integrated system of health service delivery, lessons learned from this case study can be adopted by policymakers, managers, implementation partners and implementers to inform the meaningful use of health information in policy development and implementation. A better understanding of the complex role of health information in the health system, and the circumstances under which health information can effectively be used by different health actors, can assist with preparing primary health facilities in South Africa for the roll-out of the NHI and Universal Health Coverage.

## Footnotes

1 Pseudonym.
